# Internal Secretion and the Thyroid Gland

**Published:** 1904-11-26

**Authors:** 


### INTERNAL SECRETION AND THE THYROID GLAND.

In spite of the large amount of work which has recently been done with reference to the most fascinating subject of internal secretion, it has hitherto been insufficient to yield much in the way of therapeutical results. This is due to the extreme difficulty experienced in conducting physiological experiments on animals, the complexity of the substances involved, and the differences in structure which exist between the organs of man and animals. As Sir Victor Horsley has said, the only satisfactory result at present which our undoubtedly increased knowledge has brought us has been in the treatment of myxœdema by means of thyroid extract. In this disease, although the exact way in which the deficiency of the thyroid secretion produces the well-known chain of symptoms cannot be said to be understood, the facts that the colloid substance in the thyroid is diminished and that the symptoms disappear where an extract of the gland is exhibited are thoroughly established. In the condition known as Graves' disease, the problem is more complex. In the first place the thyroid consists of two distinct tissues, the thyroid tissue proper, and the parathyroid tissue, which in man and monkeys is scattered throughout the gland, much as Langerhans's cells are scattered throughout the pancreas; though in some animals, such as dogs and rabbits, it is collected into separate masses forming distinct parathyroid glands. In exophthalmic goître the true thyroid tissue is altered, and the colloid substance disappears, and in some cases it has been shown that an increase in the amount of parathyroid tissue occurs, and at any rate morphological changes in the secreting cells take place. A similar change, namely a conversion of thyroid into parathyroid tissue was observed by Edmunds as a result of partial thyroidectomy in man, and after removal of the parathyroids in animals. It would seem, therefore, that the symptoms of exophthalmic goître are not due merely to an increase in the amount of thyroid secretion, but to a perversion of that secretion, and hence the disease is not strictly the antithesis of myxœdema. The symptoms produced in animals by the extirpation of the thyroids have been shown by MacCallum to be due mostly to the concomitant extirpation of the parathyroids, whose function is possibly to neutralise toxic substances produced elsewhere in the body; these symptoms do not in the least resemble exophthalmic goître; and, in fact, the parathyroids are not considered by him to have any marked share in the causation of that disease. On the other hand, symptoms of exophthalmic goître have been produced by large doses of thyroid extract, and the view seems feasible that the condition is primarily not due to the thyroid, but to some other organ, changes in which call for extraordinary activity in thyroid secretion with subsequent alteration in that secretion and the elaboration of a toxic substance. Thus, not only is the normal secretion absent in a well developed case, but toxic products are being manufactured in its place. Sir Victor Horsley brings forward two clinical observations in support of this view of the double nature of the changes in exophthalmic goître. In the first place, he says, when the cases were first operated on, many patients died with the same symptoms which follow thyroidectomy in a carnivorous animal, and which appear to be due therefore to athyroidism. In the second place, the toxic symptoms which are produced by adenomata of the thyroid, when presumably the secretion is altered and toxic, are relieved at once by operative measures. The theory that exophthalmic goître is a disease of the nervous system must be discarded, at least in its original form, as a result of recent investigations. It is, however, quite possible that some nervous disturbance is primarily responsible for the perversion of the thyroid secretion to which the symptoms of the disease are due.

The functions of the thyroid gland are not easily determinable, but the absence of symptoms after the operation for malignant disease, which mostly occurs in elderly persons, seems to point to some connection between thyroid secretion and the period of activity and growth of the organism. Probably, however, more careful analysis of the various results of disease may give some clue to the normal functions of this obscure and interesting structure.

